# Purification and identification of a polysaccharide from medicinal mushroom *Amauroderma rude* with immunomodulatory activity and inhibitory effect on tumor growth

**DOI:** 10.18632/oncotarget.4397

**Published:** 2015-06-27

**Authors:** Honghui Pan, Yuanyuan Han, Jiguo Huang, Xiongtao Yu, Chunwei Jiao, Xiaobing Yang, Preet Dhaliwal, Yizhen Xie, Burton B. Yang

**Affiliations:** ^1^ Guangdong Institute of Microbiology, State Key Laboratory of Applied Microbiology Southern China, Guangdong Provincial Key Laboratory of Microbial Culture Collection and Application, Guangdong Open Laboratory of Applied Microbiology, Guangzhou, China; ^2^ Yuewei Edible Fungi Technology Co. Ltd., Guangzhou, China; ^3^ Sunnybrook Research Institute, Sunnybrook Health Sciences Centre, Toronto, Canada; ^4^ Department of Laboratory Medicine and Pathobiology, University of Toronto, Toronto, Canada

**Keywords:** medicinal mushroom, herbal medicine, tumor growth, cytokine, amauroderma

## Abstract

Medicinal mushrooms in recent years have been the subject of many experiments searching for anticancer properties. We previously screened thirteen mushrooms for their potential in inhibiting tumor growth, and found that the water extract of *Amauroderma rude* exerted the highest activity. Previous studies have shown that the polysaccharides contained in the water extract were responsible for the anticancer properties. This study was designed to explore the potential effects of the polysaccharides on immune regulation and tumor growth. Using the crude *Amauroderma* rude extract, *in vitro* experiments showed that the capacities of spleen lymphocytes, macrophages, and natural killer cells were all increased. *In vivo* experiments showed that the extract increased macrophage metabolism, lymphocyte proliferation, and antibody production. In addition, the partially purified product stimulated the secretion of cytokines *in vitro*, and *in vivo*. Overall, the extract decreased tumor growth rates. Lastly, the active compound was purified and identified as polysaccharide F212. Most importantly, the purified polysaccharide had the highest activity in increasing lymphocyte proliferation. In summary, this molecule may serve as a lead compound for drug development.

## INTRODUCTION

As a great source of polysaccharides, medicinal mushrooms are commonly used herbs in oriental countries for treatment of various diseases. A variety of pharmaceutical properties of polysaccharides have been revealed including antioxidant [1], anti-inflammatory [2], antiallergic [3], antitumor [4–6], antidiabetes [7], anticoagulant [8], antiviral [9], immunomodulatory [10], antihepatopathy [11], and antifatigue [12]. The polysaccharides contained in some mushrooms have been shown to have immonopotentiating effects on many cells in the immune system, including T cells, B cells, and macrophages [13]. For example, Polysaccharide Krestin (PSK) and Polysaccharide Peptide (PSP), isolated from *Coriolus versicolor*, have been subject to clinical trials, and shown to have positive effects against cancer, and they have been used in clinical settings for several decades [14, 15]. Furthermore, the extraction and use of polysaccharides from many other mushrooms such as *Ganoderma lucidum* [16] *Lentinula edodes* [17], *Agaricus blazei* [18], *Antrodia camphorate* [19], and *Grifola frondosaI* [20] have also been shown to stimulate immune activity. This caused us to look further into another mushroom, *Amauroderma rude*.

*Amauroderma rude* (Berk.) Torrend belongs to the Ganodermataceae family and distributes in the tropical and subtropical zone [21]. We have previously demonstrated that the water extract of *Amauroderma rude* can inhibit cancer cell growth [6]. Since polysaccharides may be the major components in the water extract, we hypothesized that the polysaccharides of *Amauroderma rude* may have immune regulatory activity. This study was designed to explore the potential effects of the polysaccharides isolated from *Amauroderma rude* on stimulating immune activity and tumor growth inhibition.

## RESULTS

### *Amauroderma rude* extract increased the immunomodulatory activity *in vitro*

**Figure 1:**
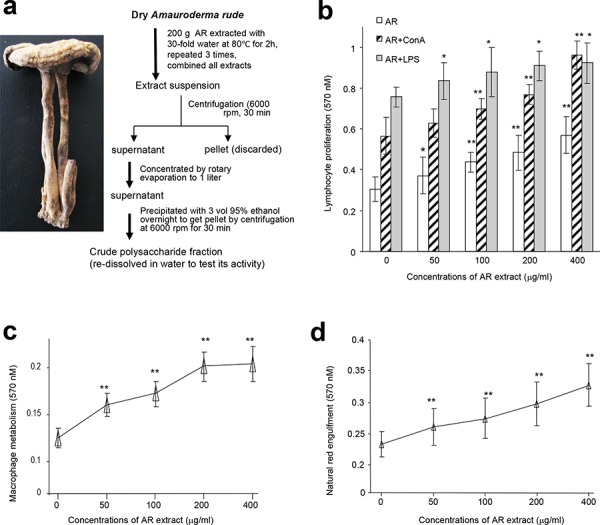
Effects of AR on cellular activities of mouse spleen lymphocytes and macrophages **a.** Procedure for preparation of water extract from *Amauroderma rude*. **b.** Mouse spleen lymphocytes were incubated with AR extract at 37°C for 44 h, followed by MTT assay. Incubation with AR extract increased lymphocyte proliferation. Inclusion of ConA and LPS displayed additive effect on enhancing lymphocyte proliferation. Asterisks indicate significant differences. *p < 0.05, **p < 0.01. Error bars, SD (*n* = 5). All experiments were repeated three times. **c.** In energy metabolism assay, mouse macrophages were incubated at 37°C for 2 h, followed by addition of drugs or AR extract. The cultures were then incubated at 37°C for 20 h followed by MTT assays. Treatment with AR extract improved energy metabolism. Asterisks indicate significant differences. **p < 0.01. Error bars, SD (*n* = 5). **d.** In the neutral red engulfment assay, cells in the 96-well plates were incubated at 37°C for 24 h. After centrifugation (1800 rpm, 5 min), 100 μl of neutral red in physiological saline solution (0.1%) were added to each well. The plates were incubated at 37°C for 30 min. After centrifugation (1800 rpm, 5 min), each well was washed with 200 μl PBS twice, followed by addition of 100 μl cytolysate (acetic acid:anhydrous alcohol = 50:50). The cells were incubated at room temperature overnight followed by MTT assay. Incubation with AR extract increased engulfment of neutral red by the cells. Asterisks indicate significant differences. **p < 0.01. Error bars, SD (*n* = 5).

We examined the possible immunomodulatory activity of the crude polysaccharides from the water extract of *Amauroderma rude* (AR). Mouse lymphocytes were isolated from the spleen and their proliferation was tested in the presence of the water extract, along with endotoxins, lipopolysaccharides (LPS), and Concanavalin A (ConA), since proliferation of spleen lymphocytes is the prime indicant of immunopotentiation [13]. We found that AR extract was the most effective agent in stimulating proliferation of the lymphocytes (Figure 1a and 1b). AR alone or acting together with LPS/ConA significantly increased lymphocyte proliferation in a dose-dependent manner compared with the control group.

**Figure 2:**
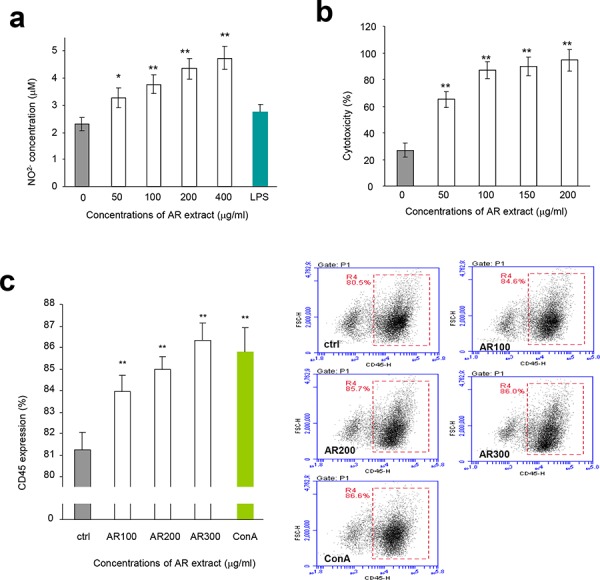
Effects of AR on macrophages and natural killer cells **a.** Mouse macrophages were incubated at 37°C for 2 h. Drugs and samples were added to the cultures at the concentrations indicated. The cultures were incubated at 37°C for 48 h followed by Griess assay to measure nitric oxide concentrations. Incubation with AR extract increased the production of nitric oxide by macrophages. Asterisks indicate significant differences. *p < 0.05, **p < 0.01. Error bars, SD (*n* = 5). All experiments were repeated three times. **b.** In NK cell assay, the harvested lymphocytes were incubated at 37°C for 2 h followed by treatment with AR extract. The effect of NK cells was assayed as described in the Methods. Incubation with AR extract stimulated and increased the activity of NK cells in killing breast cancer cells. Asterisks indicate significant differences. *p < 0.05, **p < 0.01. Error bars, SD (*n* = 5). **c.** Lymphocytes were inoculated and incubated at 37°C for 2 h, followed by addition of drugs and samples. The cultures were then incubated at 37°C for 48 h before flow cytometry analysis for CD45 expression. Incubation with AR extract increased CD45 expression. Asterisks indicate significant differences. *p < 0.05, **p < 0.01. Error bars, SD (*n* = 5). Right, typical measurements of CD45 levels by flow cytometry.

We also isolated macrophages from mouse peritoneal cavity and incubated them with AR, since macrophages exert a variety of complex microbicidal functions [22]. We found that incubation with AR improved energy metabolism of macrophages (Figure 1c) and increased macrophage engulfing of neutral red (Figure 1d). Incubation of macrophages with AR also increased production of nitric oxide in a dose-dependent manner compared with the control group (Figure 2a). Natural killer (NK) cells are important effectors in innate immunity, but also play a role in the regulation of the adaptive immune response [23]. We found that incubation with AR increased the function of mouse natural killer cells significantly, by destroying more cancer cells (Figure 2b). CD45 is a cell surface glycoprotein that has been implicated in the integrin-mediated adhesion of macrophages, and is reported to affect the functional responsiveness of cells to chemo-attractants [24]. We found that incubation with AR increased the percentage of CD45 significantly, compared with the control group (Figure 2c).

### *Amauroderma rude* extract increased immunomodulatory activity *in vivo*

**Figure 3:**
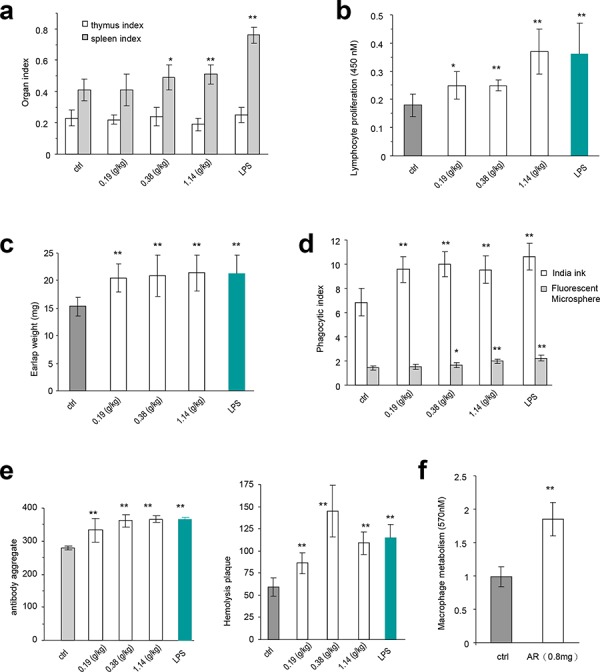
The immunomodulatory activity of *Amauroderma rude* in normal animals **a.** The *in vivo* experiments were described in detail in the Methods. Injection of normal mice with AR extract increased sizes of spleens, but had little effect on thymus. Similarly, injection with LPS only increased the size of spleen but not the thymus. Asterisks indicate significant differences. *p < 0.05, **p < 0.01. Error bars, SD (*n* = 5). All experiments were repeated three times. **b.** Injection of mice with AR extract increased the proliferation rates of the spleen lymphocytes. Asterisks indicate significant differences. *p < 0.05, **p < 0.01. Error bars, SD (*n* = 5). **c.** Mice injected with AR extract displayed increased weight of the earlaps. Asterisks indicate significant differences. *p < 0.05, **p < 0.01. Error bars, SD (*n* = 5). **d.** Engulfment of Fluorescent Microspheres and Indian ink by the cells was increased by treatment with AR extract. Asterisks indicate significant differences. *p < 0.05, **p < 0.01. Error bars, SD (*n* = 5). **e.** Antiserum isolated from mice injected with AR extract displayed hemolysis plaques (left) and antibody aggregation in the agglutination assay (right). Asterisks indicate significant differences. *p < 0.05, **p < 0.01. Error bars, SD (*n* = 5). **f.** Injection of mice with AR extract increased energy metabolism of macrophages. Asterisks indicate significant differences. *p < 0.05, **p < 0.01. Error bars, SD (*n* = 5).

We explored the immunomodulatory activity of *Amauroderma rude* in normal mice that maintain a normal immune system. The effect of AR on the spleen and thymus was examined by evaluating the spleen index and thymus index of five groups. We found that the spleen index of mice treated with AR extract increased significantly compared to the control group, while the thymus index was not affected (Figure 3a).

We then examined the immunomodulatory activity of AR in cellular immune function, humoral immunity, and mononuclear macrophage function. The spleen lymphocyte proliferated faster in all groups treated with AR extract, although not to the levels of LPS treatment, compared with the negative control group (Figure 3b). The weight of earlaps increased significantly in the mice treated with AR extract and LPS (Figure 3c).

Mononuclear macrophages were isolated from mice injected with AR extract and LPS. Their function was assessed using the carbon clearance test and engulfment of fluorescent microspheres. We evaluated the phagocytic index of AR groups and found significant increase with all doses of AR extract in the carbon clearance test, compared to the negative control group (Figure 3d). The fluorescent microsphere index reached significant levels only when the AR doses were relatively high (Figure 3d).

In the humoral immunity assay, we detected a significant increase in the aggregation of antibodies (Figure 3e, left), and the formation of hemolysis plaques (Figure 3e, right), when the mice were treated with AR extract or LPS. In the energy metabolism assay, we detected significant increase in the proliferation of macrophages isolated from mice injected with AR extract (Figure 3f).

**Figure 4:**
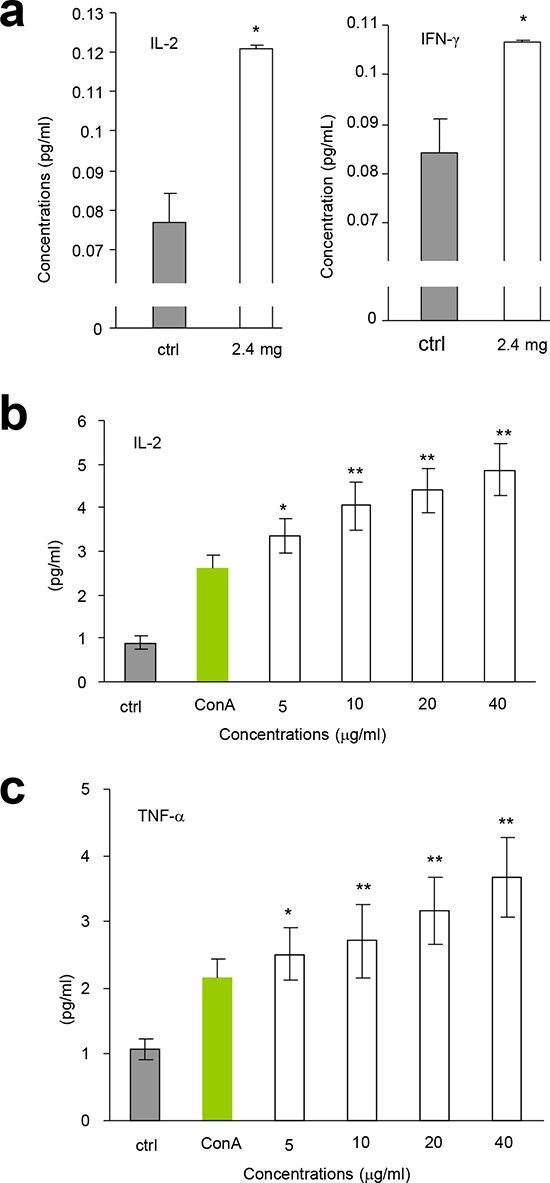
AR induced cytokine expression **a.** After tumor cell implantation, the mice were orally given AR extract (2.4 mg/mouse). 7 h later, the levels of secreted IL-2 (left) and IFN-γ (right) were measured. Asterisks indicate significant differences. *p < 0.05, Error bars, SD (*n* = 5). All experiments were repeated three times. **b-c.** AR extract was applied to the cultures of mouse spleen lymphocytes in the concentrations indicated. 24 h later, the levels of secreted IL-2 (b) and TNF-α (c) were measured. Asterisks indicate significant differences. *p < 0.05, **p < 0.01. Error bars, SD (*n* = 5).

We tested the immunoreactivity of the partially purified polysaccharides *in vivo*. After tumor cell implantation, the mice were orally given the polysaccharide fraction with the highest activity in lymphocyte proliferation (Fraction F_0.5_, 2.4 mg/mouse). 7 h after delivery, we detected significant increase in the secretion of IL-2 (Figure 4a, left) and IFN-γ (Figure 4a, right) compared with the control. These results were further confirmed by *in vitro* assays, where the partially purified F_0.5_ Fraction was applied to mouse spleen lymphocytes. 24 h after the addition of the polysaccharides, we detected significant increase in the secretion of IL-2 (Figure 4b) and TNF-α (Figure 4c) in a dose-dependent manner, compared to the control.

### *Amauroderma rude* extract inhibited tumor growth in mice

**Figure 5:**
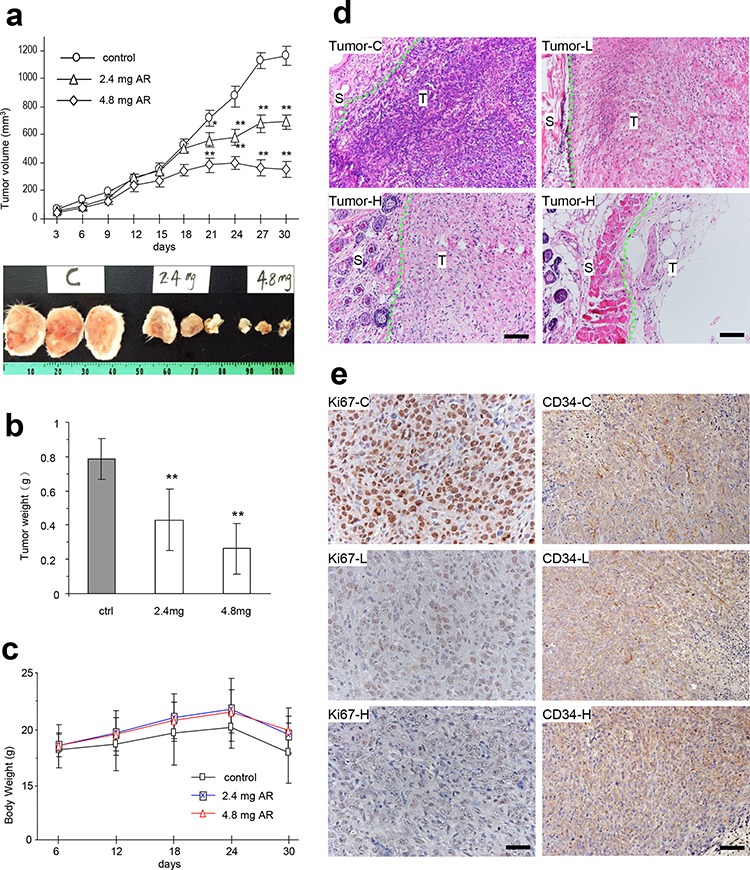
AR inhibited tumor growth by inducing tumor cell death and inhibiting Ki67 and CD34 expression **a-b.** After tumor cell implantation, the mice were injected with AR extract and monitored for tumor growth. Injection with AR extract inhibited tumor growth by decreasing tumor volumes (a, upper, tumor growth curve, lower, typical photo of tumor sizes) and decreased tumor weight (b). Greater dose of AR extract displayed stronger inhibitory effect on tumor growth. Asterisks indicate significant differences. *p < 0.05, **p < 0.01. Error bars, SD (*n* = 12 mice). All experiments were repeated three times. **c.** The injection of AR had little effect on the body weight of the mice. **d.** Tumor sections were subject to H&E staining and examined under a light microscope. Extensive cell death was detected in mice injected with both doses of polysaccharides. Tumor-C, control group; Tumor-L, low dose group; Tumor-H, high dose group; S, stromal tissue; T, tumor; Scale bars, 50 μm. **e.** Tumor sections (control, low dose, and high dose) were subject to Ki67 and CD34 staining followed by examination and photograph under a light microscope. Tumors formed in the mice injected with the polysaccharides, and displayed a decreased number of Ki67 positive cells and CD34 positive cells. Scale bars, 50 μm.

We then explored whether injection of the AR extract affected tumor growth. After tumor implantation, the mice were injected with AR extract and the tumor growth rates were monitored every three days. We detected a significant decrease in tumor growth rates starting on day 20 in both groups, which were injected with two doses of AR extract (Figure 5a, upper for tumor growth rates, lower for a photo of typical tumor sizes). When the animal experiment finished, the tumors were excised and weighed. The weight of tumors decreased significantly in both groups of mice that were injected with AR extract (Figure 5b). The average body weight of the mice injected with AR extract was higher compared to the control (Figure 5c). This suggests improved quality of life.

Tumor sections were subject to H&E staining and examined under a light microscope. We found that the AR extract induced extensive tumor cell death (Figure 5d). We also examined the proliferative capacity of the tumor cells using Ki67 positive cells. Injection of AR extract in the tumor implanted mice significantly decreased Ki67 positive cells, which suggested decreased proliferative activity (Figure 5e). Furthermore, CD34 staining also displayed decreased levels of positive cells in these mice (Figure 5e).

### The partially purified *Amauroderma rude* extract activated mouse immune activity and inhibited tumor growth

**Figure 6:**
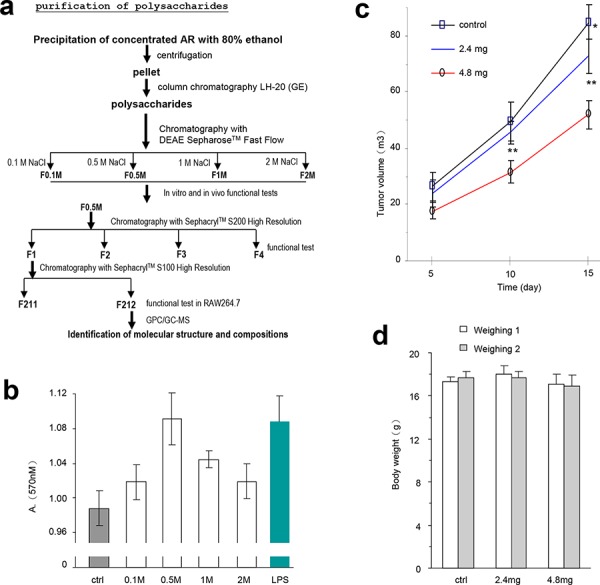
Partial purification of the polysaccharides and their effects on lymphocyte activity and tumor growth **a.** Procedure for purification of polysaccharides from the water extract of *Amauroderma rude*. **b.** The effect of four different components purified from AR extract on the proliferation of mouse spleen lymphocytes. It appeared that the fraction eluted with 0.5 M (F_0.5_) NaCl displayed the strongest effect on increasing lymphocyte proliferation. **c.** After tumor cell implantation, the mice were injected with the partially purified polysaccharides F_0.5_ at different time points as indicated in the Methods section, and monitored for tumor growth. Injection with F_0.5_ inhibited tumor growth in both doses used. Asterisks indicate significant differences. *p < 0.05, **p < 0.01. Error bars, SD (*n* = 12 mice). **d.** The body weight of the mice was not affected by tumor cell implantation or by polysaccharide injection.

To understand the components that played a role in the modulation of immune activity and inhibition of tumor growth, we purified the AR extract using column fractionation (Figure 6a). The effects of different fractions of the partially purified polysaccharides were tested by lymphocyte proliferation. Mouse spleen lymphocytes were used, alongside LPS as the positive control. The experiment showed that the F_0.5_ was the most effective fraction among the four fractions tested. It increased lymphocyte proliferation to a level similar to LPS (Figure 6b).

The effect of the partially purified polysaccharides on tumor growth was tested. We delivered the F_0.5_ fraction into mice that had been implanted with tumor cells. F_0.5_ inhibited tumor growth significantly (Figure 6c), but had no effect on the size of the mice (Figure 6d). These results suggest that the polysaccharides of *Amauroderma rude* exerted two layers of effects on tumor growth *in vivo*: it inhibited tumor cell proliferation and it increased tumor cell death.

### The purified *Amauroderma rude* polysaccharide promoted mouse immune activity

**Figure 7:**
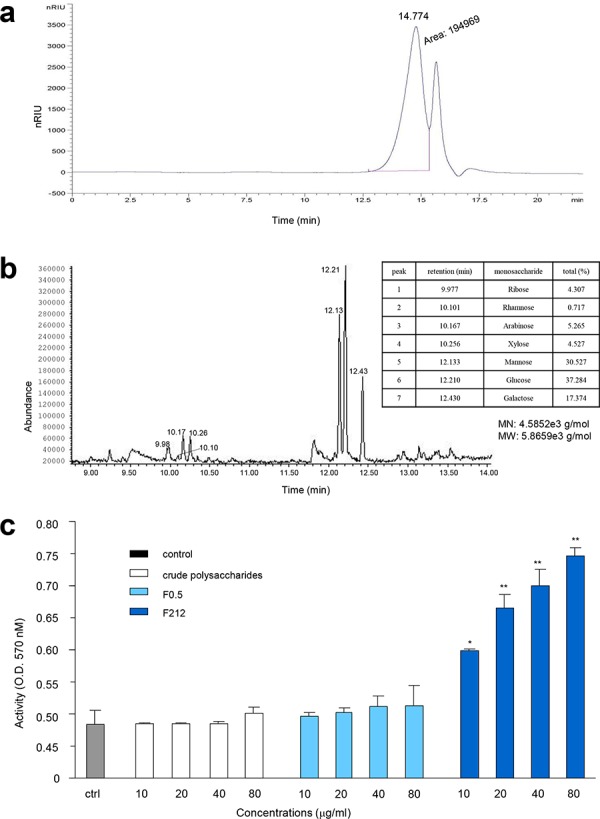
Purification of polysaccharides and their effects on cell proliferation GC-MS **a.** Diagram showing two components (F211 and F212) after chromatography from F1 fraction using SephacrylTM S100 High Resolution. **b.** Identification of the molecular structure and composition of F212. The peaks were of polysaccharide characteristics, and are labeled with numbers. The table inset provides the names and composition of the monosaccharides. The molecular number and molecular weight of the purified polysaccharide are shown underneath the table. **c.** Incubation of the purified polysaccharide (F212) with mouse cell line RAW264.7 increased cell proliferation. At the concentrations used, the AR crude extract and the partially purified polysaccharides (F0.5) showed little effect on cell proliferation as compared with the control (ctrl). Asterisks indicate significant differences. *p < 0.05, **p < 0.01. Error bars, SD (*n* = 5).

We further purified the fraction F_0.5_ by chromatography using column Sephacryl™ S200 High Resolution. Four fractions were obtained (Figure 6a, F1, F2, F3, and F4). We identified that F1 possessed the highest activity in functional tests. This fraction was further purified by chromatography using column Sephacryl™ S100 High Resolution. One typical peak of polysaccharides was detected (Figure 7a, labeled 14.774). After functional testing, we detected major activity in the peak of F212. We identified the monosaccharide compositions of F212 by contrasting monosaccharides with reference substances and using the Total Ion Chromatogram (TIC) (Figure 7b). Data analysis indicated that F212 was made up of Ribose, Rhamnose, Arabinose, Xylose, Mannose, Glucose, and Galactose, respectively (Figure 7b, table inset). A larger view of the graph is provided in Supplementary Figure S1. The molecular weight of the purified polysaccharide was calculated (MN: 4.5852e3 g/mol, MW: 5.8659e3 g/mol).

Finally, we tested the role of the purified polysaccharide F212 in cell proliferation. Incubation of F212 with mouse cell line RAW264.7 increased their proliferation (Figure 7c). However, at the concentrations used, the AR extract and the partially purified polysaccharides showed little effect on cell proliferation compared to the control.

## DISCUSSION

We previously reported that the water extract of *Amauroderma rude* possessed the highest activity among thirteen different medicinal mushrooms tested in inducing cancer cell death [6]. It is believed that the polysaccharides isolated from medicinal mushrooms are the major components that possess anticancer activities [25–27]. In this study, we precipitated the polysaccharides from the water extract of *Amauroderma rude* using a high concentration of ethanol. After a series of chromatography purification, we obtained a single peak (F212) and identified the monosaccharide components of the polysaccharide.

Polysaccharides have been extensively isolated from many medicinal mushrooms and their functions have been studied showing anticancer properties [28–31]. The polysaccharide we purified represented the first polysaccharide from *Amauroderma rude*, and it showed to have strong functions in immunopotentiation *in vitro* and *in vivo*. We found that the crude extract was able to increase lymphocyte proliferation, increase natural killer cell activity, and increase macrophage metabolism and phagocytosis. Consistent with these effects, CD45 expression, production of NO, and production of antibodies were also up-regulated. Our results were consistent with another mushroom, *Ganoderma lucidum*, whose isolated polysaccharides, small molecules, and total extract showed many of the same anticancer effects [32–37]. As a result of the strong immunoregulatory activity, we detected a significant inhibitory effect of *Amauroderma rude* on tumor growth. Moreover, the *Amauroderma rude* extracts were injected in areas far away from the tumor implantation sites in the mice. Therefore, this suggests that *Amauroderma rude* caused the inhibitory effect on tumor growth by regulating the immune system.

We tested the hypothesis that *Amauroderma rude* could regulate the immune activity of the host by stimulating the production of cytokines. It has been reported that the polysaccharides can promote expression of tumor necrosis factor alpha (TNFα) and interferon gamma (IFNγ) [38]. TNFα is a cytokine involved in systemic inflammation, which is expressed mainly by activated macrophages, lymphocytes, and NK cells. TNFα can promote an inflammatory response and exert an acute immune response [39, 40]. IFNγ is a dimerized cytokine that is critical for innate and adaptive immunity against infections. It is an important activator of macrophages and can be secreted by NK cells and macrophages. Therefore it also has immunoregulatory and antitumor properties [41–43]. Our results showed that treatment with *Amauroderma rude* induced significant production of TNFα and IFNγ. This may represent the essential role of *Amauroderma rude* in the inhibition of tumor growth. In clinical studies, cancer patients in advanced stages were given polysaccharide fractions of *Ganoderma lucidum* orally three times a day for 12 weeks. This treatment resulted in a significant increase in the plasma concentrations of IL-2, IL-6, and IFNγ, and a significant increase in the mean NK cell activity compared to baseline [44]. Likewise, our *in vitro* and *in vivo* experiments showed that *Amauroderma rude* also stimulated the production of IL-2, TNF-α and IFNγ. IL-2 is a cytokine signaling molecule in the immune system where it regulates the activities of white blood cells (e.g. lymphocytes) that are responsible for the immunity [45]. Our results showed that the polysaccharides from *Amauroderma rude* may function similarly to those from *Ganoderma lucidum*, by producing these cytokines with potent effects on immunity and anticancer.

To test the effect of polysaccharides on regulating immune activity and inhibiting tumor growth, it is essential to perform the experiments in normal mice that have normal immune systems. In cancer research, most of the tumor growth experiments are conducted in nude mice using human cancer cell lines. Nude mice have deficient immune systems to begin with, and are not ideal animals to study drugs and herbal medicine which may possess immune-regulatory activity. Thus, a tumor cell line originally developed from the same strain of mice is essential to be able to form tumors in those mice.

Although we found that the F212 polysaccharide displayed immune regulatory activity, other molecules in the pool may also possess activity in immune regulation and antitumor growth. For example, when we used the chromatography approach to purify the bioactive ingredients, we could only use the fraction with the highest immune regulatory activity for further purification. This relied on the total activity of each fraction, rather than the activity of a single molecule. It is possible that one fraction may contain some molecules with very high immune regulatory activity, but still not be selected because the fraction contained fewer such molecules. Moreover, other fractions that were not selected could contain novel molecules with potent immune regulatory activity. This awaits further investigation.

In general, it is known that large molecules with complex structures possess strong capacities to induce immune responses. For example, large polysaccharides linking to a peptide, such as PSK and PSP purified from *Coriolus versicolor* [14, 15], and some complex polysaccharides purified from *Ganoderma lucidum*, have been found to have a strong effect on immunity. An unexpected result from our study was the identification of a relatively small polysaccharide F212 that also possessed strong immunity. The composition of F212 appeared similar to many polysaccharides purified from other medicinal mushrooms. The structure of F212 awaits future investigation.

In summary, we have purified and identified a small polysaccharide with strong immunity from a newly recognized medicinal mushroom, *Amauroderma rude*. This represents the first known polysaccharide of this species, which has strong anticancer activity [6]. It appears that polysaccharides from *Amauroderma rude* possess multiple effects on regulating the immune system at the molecular and cellular level. This may explain the strong immune function of the molecule. Understanding the structure and function of this molecule may provide helpful information in drug design.

## MATERIALS AND METHODS

### Preparation of the aqueous extract from *Amauroderma rude*

We extracted the aqueous fraction from *Amauroderma rude* fruit bodies which were supplied by Yuewei Edible Fungi Technology Co. Ltd and authenticated for utilization by the Center for Research and Development of Edible Fungi, Guangdong Institute of Microbiology.

Dry *Amauroderma rude* fruit bodies (200 g) were crushed and extracted with 30-folds of water at 80°C for 2 h. After centrifugation (6000 rpm, 30 min), the supernatants were collected and the precipitation was extracted another two times. All supernatants were concentrated to 1 liter and precipitated with 3 volumes of 95% ethanol overnight, followed by centrifugation (6000 rpm, 30 min). The residue ethanol and water was removed at 50°C. The yield of crude polysaccharide fraction (AR) was 2.1% of the fresh weight.

### Purification of crude polysaccharides

Column fractionation was the process used to purify the biologically active ingredient of AR. AR was suspended with distilled water. In the meantime, 750 ml DEAE Sepharose Fast Flow was packed into a column of 15 × 70 cm (d × h).

The AR suspension was applied to the top of the Sepharose gel bed. After twenty min of careful application of the sample, the column was eluted with four bed volumes of 0.1, 0.5, 1.0, and 2.0 mol of NaCl at an elution speed of 20 ml/min. Each collection contained 1500 ml. Every collection was concentrated and lyophilised producing fractions F_0.1_, F_0.5_, F_1_, and F_2_. The immunomodulatory activity was screened by proliferation of mouse spleen lymphocytes.

### Proliferation and CD45 expression of mouse lymphocytes from spleen

Male BALB/c mice (5-week old, 18 to 20 g) were purchased from Guangdong Medical Laboratory Animal Center (Foushan City, China). The license number was SCXK2008-0002. The health certificate number was 44007200005061. The animals were provided with water and mouse chow *ad libitum*, and were housed in a rodent facility at 22 ± 1°C with a 12 h light-dark cycle for acclimatization. All procedures involving animals and their care were approved by the Ethics Committee of the Guangdong Laboratory Animals Monitoring Institute. Our previous results proved the LD50/(mg/kg) of AR was above and beyond 15000 mg/kg and AR was regarded as nontoxic.

The mice were sacrificed by dislocating cervical vertebrae and marinated in 75% ethanol for three min. The spleen was excised from the mice in sterile environment and rinsed with serum-free RPMI-1640 medium twice, then grinded and passed through a 100-mesh sieve. The suspended lymphocytes were pelleted by centrifugation (1000 rpm, 5 min), resuspended in Tris-NH_4_Cl and pelleted by centrifugation (1000 rpm, 5 min) to remove red blood cells. This step was repeated once and the cells were resuspended in serum-free RPMI-1640 to wash one more time. After being pelleted by centrifugation (1000 rpm, 5 min), the cells were resuspended in RPMI-1640 medium and the number of cells were counted. The cells (5 × 10^6^ cells/ml) were seeded on 96-well tissue culture plates in RPMI-1640 containing 10% FBS. The plates were incubated at 37°C for 2 h. Drugs and samples were added to the cultures at the concentrations indicated in each figure. The cultures were subject to MTT assay after incubating at 37°C for 44 h.

For measurement of CD45 production, the lymphocytes were divided into five groups: control, ConA (5 μg/ml), AR-100 (5 μg/ml), AR-200 (5 μg/ml), and AR-300 (5 μg/ml). Cells (5 × 10^6^ cells/ml) were seeded on 24-well tissue culture plates in RPMI-1640 containing 10% FBS. The plates were incubated at 37°C for 2 h, followed by addition of drugs and samples. The cultures were then incubated at 37°C for 48 h before flow cytometry analysis for CD45 expression. The procedure was performed as described [46].

### Assays using mouse peritoneal macrophages

Male BALB/c mice (5-week old, 18 to 20 g) were sacrificed by dislocating cervical vertebrae and marinated in 75% ethanol for three min. Serum-free RPMI-1640 medium was injected into the peritoneal cavity and withdrawn to collect the ascites. After centrifugation (1000 rpm, 5 min), cells in the pellets were suspended in RPMI-1640 and the number of cells were counted. Cells (5 × 10^6^ cells/ml) were seeded on 96-well tissue culture plates in RPMI-1640 containing 10% FBS.

In energy metabolism assay, the plates were incubated at 37°C for 2 h. Drugs and samples were added to the cultures at the concentrations indicated in each figure. The cultures were then incubated at 37°C for 20 h followed by a MTT assay.

In the neutral red engulfment assay, cells in the 96-well plates were incubated at 37°C for 24 h. After centrifugation (1800 rpm, 5 min), 100 μl of neutral red in physiological saline solution (0.1%) were added to each well. The plates were incubated at 37°C for 30 min. After centrifugation (1800 rpm, 5 min), each well was washed with 200 μl PBS twice, followed by addition of 100 μl cytolysate (acetic acid:anhydrous alcohol = 50:50). The cells were incubated at room temperature overnight followed by MTT assay.

To measure the nitric oxide concentrations, the plates were incubated at 37°C for 2 h. Drugs and samples were added to the culture at the concentrations indicated in each figure. The cultures were measured after incubating at 37°C for 48 h by Griess assay.

### Assay using natural killer (NK) cells

In NK cell assay, the harvested mixture in the lymphocytes was suspended in Tris-NH_4_Cl and centrifuged (1000 rpm, 5 min), repeated twice. The pellets were then suspended in serum-free RPMI-1640 and centrifuged (1000 rpm, 5 min). The pellets were resuspended with RPMI-1640 and the number of cells were counted. Cells (5 × 10^6^ cells/ml) were seeded on 96-well tissue culture plates in RPMI-1640 supplemented with 10% FBS. The plates were incubated at 37°C for 2 h. Drugs and samples were added to the cultures. The effect of the NK cells was assayed after incubating at 37°C for 24 h as follows: human breast cancer cells MT-1 (1 × 10^8^ cells/ml) were used as target cells and incubated with three groups of NK cells: group I: 100 μl NK cells mixed with 100 μl MT-1 cells, group II: 100 μl NK cells mixed with 100 μl RPMI-1640, and group III: 100 μl MT-1 cells mixed with 100 μl RPMI-1640. The plates were incubated at 37°C for 20 h followed by MTT assay.

### Effect of AR on macrophages *in vivo*

For energy metabolism assay, male BALB/c mice (5-week old, 18 to 20 g) were divided into two groups. One group of mice was lumbar injected with 200 μl AR (4 mg/ml), and the other group served as a control. 24 h after the injection, proliferation of peritoneal macrophages was measured by MTT assay as described earlier.

In phagocytic index assay, the function of the macrophages was assessed via a carbon clearance test. Each mouse was intravenously injected with diluted India ink at 100 μl/10 g body weight. Blood specimens were collected at 2 min (t1) and 10 min (t2) from the retinal venous plexuses, and 20 μl blood was then mixed with 2 ml 0.1% Na_2_CO_3_. The absorbance at 600 nm was measured on a UV-visible spectrophotometer with 0.1% Na_2_CO_3_ as the blank. The liver and the spleen were weighed, and the phagocytic index was calculated as follows:

K = (lg OD1 − lg OD2)/(t2 − t1), where OD1 was for t1 and OD2 was for t2.

Phagocytic index ˛ $=\sqrt[{3}]{\kappa }\times \text{A}/(\text{B}+\text{C})$, where A is the body weight, B is the liver weight, and C is the spleen weight.

### Effect of AR on the immunoregulation *in vivo*

Female BALB/c mice (5 week old, 18 to 20 g) were randomly divided into 4 groups (12 mice each). The mice were given the crude polysaccharides orally (0.2 ml/mouse, low dose, 19 mg/ml, medium dose, 38 mg/ml, and high dose, 114 mg/ml) every two days for a total of 30 days. The negative control group contained water alone. The positive control group was lumbar injection with LPS, every other day for five times totally (0.1 ml for the first time and 0.2 ml each for the following 4 times, 0.05 mg/ml).

The mice were subject to visceral organ weight measurements. 24 h after the last drug administration, the animals were weighed and then sacrificed via decapitation. The spleen and thymus were excised. The spleen, thymus, and body weight of every group were measured. Organ index (mg/g) = (weight of thymus or spleen)/body weight.

For lymphocyte proliferation assay, the spleens were excised sterilely from the mice 24 h after the last drug administration. Lymphocytes were prepared at a density of 5 × 10^7^ cells/ml. Cells (5 × 10^7^ cells/ml) were seeded on 96-well tissue culture plates in RPMI-1640 containing 10% FBS. The plates were incubated at 37°C for 2 h. Drugs or the AR product were added to the cultures at the concentrations indicated in the figure. The cultures were then measured after incubating at 37°C for 72 h by MTT assay.

For Delayed hypersensitivity (DH) (auricle swelling model) assay, the abdomen was shaved and covered with 50 μl DNFB 24 h after the last drug administration. The right ear was covered with 10 μl DNFB 5 days later. After 24 h, the mice were sacrificed by dislocating cervical vertebrae and the earlaps were cut. The earlaps with a diameter 8 mm were weighted.

For antibody production assay, the mice were injected in the lumbar with 0.2 ml sheep erythrocyte suspension (2%) 24 h after the last drug administration. Five days later, the spleen was taken sterilely from the mice and used to prepare a cell suspension (5 × 10^6^−1 × 10^7^ cells/ml). The activity of the antibody was tested in 6-well plates, which were pre-coated with 0.5% agarose gel (1 ml/well) dissolved in PBS. The spleen cell suspension (200 μl) was mixed with sheep erythrocyte suspension (50 μl, 20%) and 0.5 ml agarose gel (0.5% dissolved in Hank's buffer and pre-warmed to 46–50°C), followed by immediate addition to the agarose gel-coated 6-well plates. The plates were incubated at 37°C for 1 h, followed by addition of 500 μl complement (1:10 dilutions). After incubation at 37°C for 2 h, hemolysis plaques were counted. Hemolysis plaque = plaque/10^6^ spleen cells.

For Delayed Type Hypersensitivity assay, the mice were lumbar injected with 0.2 ml sheep erythrocyte suspension (2%) 24 h after the last drug administration. Five days later, serum was harvested by centrifugation. The serum was doubling diluted and added into Microhemagglutination Test Board (100 μl/well), followed by addition of sheep erythrocyte suspension cells (0.5%, 100 μl/well). The board was incubated at 37°C for 3 h. Antibody agglutination was examined and calculated as follows: Aggregate = (S1+2S2+3S3……nSn), in which 1, 2, 3……n represented dilution factors and S means the degree of agglutination.

To test engulfment of Fluorescent Microsphere, the mice were lumbar injected with 0.2 ml suspension of sheep erythrocyte (2%), 24 h after the last drug administration. Ascites were harvested by injecting 3 ml Hank's (serum containing medium) into the peritoneal cavity. Cell number was adjusted to 4–6 × 10^5^cells/ml. The cells (1 ml) were added to the 6-well tissue culture plates, to which Fluorescent Microspheres (1 × 10^7^ cells/plate) had been added. The plates were incubated at 37°C for 2 h. The supernatant was removed and 0.3 ml PBS (4°C) was added. The cells were suspended and separated from the free Fluorescent Microspheres by passing through a 200-mesh sieve. Engulfment of Fluorescent Microspheres by the cells was detected by flow cytometry. The Engulf percentage (%) and Engulf index were calculated as follows:

Phagocyticpercentage (%) = macrophage (Engulf Fluorescent Microsphere)/macrophage(total)

Phagocytic index = Fluorescent Microsphere engulfed/macrophage (total)

### Effect of AR on tumor growth

Tumor growth assay was performed as described [47, 48]. Briefly, female BALB/c mice (5 week old, 18 to 20 g) were randomly divided into 3 groups (12 mice each). 4T1 cells (2 × 10^5^ cells in 100 μl) were injected into each mouse. The third day after cell implantation, the mice were lumbar injected with drugs or test samples (0.2 ml/mouse) every three days for a total of ten injections. The test samples were either 2.4 mg/mouse (Group I), or 4.8 mg/mouse (Group II). The negative control was serum-free RPMI-1640 medium. Tumor growth was monitored twice per week. All of the mice were sacrificed by cervical dislocation and tumors were harvested on day 15. When the animal experiment finished, the body weight and tumor weight was measured.

The partially purified polysaccharides (F_0.5_) were also used to evaluate its effect on tumor growth in the same approach. Tumor growth was monitored every 5 days. The body weight was measured at the beginning and end of the experiment. The tumor sections were examined by immunohistochemical staining and expression of IL-2, TNF-α and IFN-γ were examined by ELISA test.

### Statistical analysis

The results of all the experiments were subject to statistical analysis by *t*-test. The levels of significance was set at *p* < 0.05 (*) and *p* < 0.01 (**) respectively.

## SUPPLEMENTARY FIGURE



## Abbreviations

DMEM: Dulbecco's modified Eagle's medium
FBS: fetal bovine serum
PAGE: polyacrylamide gel electrophoresis
IL-2: Interleukin 2
TNFα: tumor necrosis factor alpha
IFNγ: interferon gamma
