# Supplementary material for: Purification and identification of a polysaccharide from medicinal mushroom Amauroderma rude with immunomodulatory activity and inhibitory effect on tumor growth
Source: Oncotarget. 2015 Jun 27;6(19):17777–91. doi: 10.18632/oncotarget.4397 (PMC4627345; doi:10.18632/oncotarget.4397)
Supplement: Supplementary file 1 [file oncotarget-06-17777-s001.pdf]

SUPPLEMENTARY FIGURE

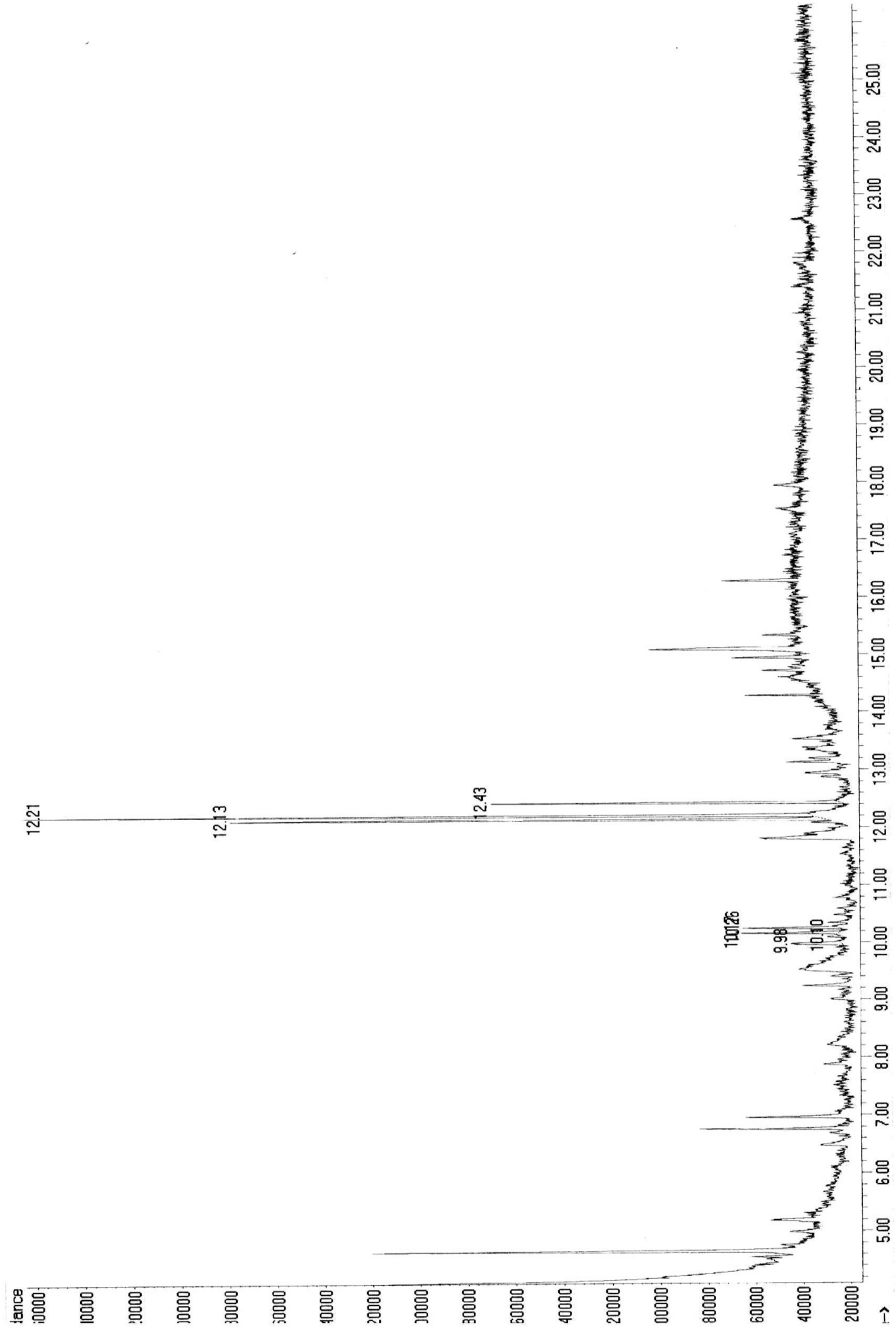

Supplementary Figure S1: The complete elution curve of polysaccharides for Fig 7b.
